# Neural networks for estimation of facial palsy after vestibular schwannoma surgery

**DOI:** 10.1007/s10877-022-00928-9

**Published:** 2022-11-04

**Authors:** Stefan Rampp, Magdalena Holze, Christian Scheller, Christian Strauss, Julian Prell

**Affiliations:** 1 Department of Neurosurgery, University Hospital Halle (Saale), Ernst-Grube Str. 40, 06120 Halle, Germany; 2 Department of Neurosurgery, University Hospital Erlangen, Schwabachanlage 6, 91054 Erlangen, Germany

**Keywords:** Facial nerve, Intraoperative monitoring, Vestibular schwannoma, Machine learning

## Abstract

**Purpose:**

Facial nerve damage in vestibular schwannoma surgery is associated with A-train patterns in free-running EMG, correlating with the degree of postoperative facial palsy. However, anatomy, preoperative functional status, tumor size and occurrence of A-trains clusters, i.e., sudden A-trains in most channels may further contribute. In the presented study, we examine neural networks to estimate postoperative facial function based on such features.

**Methods:**

Data from 200 consecutive patients were used to train neural feed-forward networks (NN). Estimated and clinical postoperative House and Brackmann (HB) grades were compared. Different input sets were evaluated.

**Results:**

Networks based on traintime, preoperative HB grade and tumor size achieved good estimation of postoperative HB grades (chi^2^ = 54.8), compared to using tumor size or mean traintime alone (chi^2^ = 30.6 and 31.9). Separate intermediate nerve or detection of A-train clusters did not improve performance. Removal of A-train cluster traintime improved results (chi^2^ = 54.8 vs. 51.3) in patients without separate intermediate nerve.

**Conclusion:**

NN based on preoperative HB, traintime and tumor size provide good estimations of postoperative HB. The method is amenable to real-time implementation and supports integration of information from different sources. NN could enable multimodal facial nerve monitoring and improve postoperative outcomes.

## Introduction

Intraoperative monitoring is applied in cerebello-pontine-angle (CPA) surgery to detect and avoid nerval damage. In vestibular schwannoma (VS) surgery, monitoring of free-running EMG, facial motor evoked potentials (MEP) and direct nerve stimulation (DNS) support preservation of facial and vestibulocochlear function and consequently postoperative quality of life [1, 2]. Monitoring of free-running EMG examines continuous EMG activity recorded by needle electrodes in the facial muscles for specific pathological patterns, so-called “A-trains”. The overall quantity of A-trains (“traintime”) has been shown to correlate with the degree of postoperative facial palsy [3, 4]. The positive predictive value of the method with fixed risk thresholds is ~ 64%, which is comparable to the values published for MEP and DNS [1, 4].

A limiting factor is the occurrence of false-positive cases with high amounts of A-trains and no severe deterioration of facial function [1, 5, 6]. In a previous study [6], we demonstrated that such patients frequently show a so-called “split” facial nerve [7]. In these cases, the intermediate nerve (NI) takes a course in the CPA separate from the facial nerve, carrying motor fibers targeting the facial muscles [6, 8–10]. Irritation of the NI provokes comparably large amounts of A-trains. Potentially due to the low functional importance of intermedius motor fibers, this is frequently not accompanied by respective deficits [6]. Unfortunately, characteristics of “intermedius” A-trains are not significantly different from “facial” A-trains [11], which prevents differentiation of the two entities. Instead, so-called A-train “clusters”, i.e. A-trains occurring in most recording channels within a short time are more frequent in patients with separate NI on a group level [11]. In addition, the observation of a separate NI increases with larger tumor size, however is rare in cases with very large tumors [11].

These findings suggest complex interactions between tumor size, NI, surgical manipulation, A-train activity and correlation to outcome. It seems therefore unsurprising that fixed traintime thresholds largely independent of tumor size and without consideration of a separate NI suffer from limitations.

In the current study, we employ machine learning and specifically neural networks (NN) to calculate an outcome parameter similar to House-Brackmann (HB) grades [12] based on traintime, tumor size and preoperative functional status. An advantage relevant to our application is the ability to integrate different data types and to capture complex interactions. While understanding the performance of a successful neural network is notoriously difficult, even a pure black-box approach may have clinical merit if it outperforms estimation based on direct interpretation of parameters alone.

The main goal of our study is therefore to provide an improved tool to estimate postoperative facial nerve outcome with the potential for real-time intraoperative application for facial nerve monitoring.

## Methods

### Patients

Data from 200 consecutive adult patients who had undergone VS surgery between 7/2006 and 8/2016 were selected retrospectively and anonymized. This study was performed in line with the principles of the Declaration of Helsinki. Approval was granted by the Ethics Committee of the University Hospital Halle (Saale) (Ref. Number 2018 − 138). All patients of whom data were included in the study had given their written informed consent for scientific usage of their data. Inclusion criteria were first VS surgery, availability of complete continuous intraoperative EMG recordings from clinical routine as well as facial nerve outcome data from follow-up after at least 6 months. Exclusion criteria were previous irradiation and neurofibromatosis.

### Recordings

Continuous EMG was recorded during the complete surgical procedure as described previously [3, 4]. In short, 15 mm long non-insulated needle electrodes were placed parallel in the facial muscles with an interelectrode distance of 5 mm. For each of the 3 main nerve branches 4 electrodes were positioned on the operated side. Referencing neighboring electrodes resulted in 3 bipolar channels per branch. The ground electrode was placed in the contralateral upper arm. Data were recorded with a Grass-Telefactor 15LT biosignal amplifier (West Warwick, RI, USA) with approximately 7 kHz and using a 5 Hz high pass filter.

### EMG processing

Recorded data was evaluated postoperatively by computer-assisted visual inspection using in-house software. Extending automated marking [3], on- and offsets of individual A-trains were marked. In addition, A-train clusters [11] were identified visually. Subsequently, the durations of all A-train events were summed up per channel, yielding a total of 9 traintime values for each patient.

### Clinical data

Clinical data were extracted from clinical documentation: preoperative and immediate postoperative facial nerve function as well as follow-up after 6 months, graded according to House-Brackmann [12]. The HB grading system distinguishes 6 degrees of facial palsy: 1 – normal function, 2 to 5 represent dysfunction from mild to severe and 6 represents total paralysis. Clinically especially relevant is HB ≥ 4 as eye closure on the affected side is no longer possible. HB degrees were checked and corrected, if necessary, by a single experienced evaluator (author JP) to reduce limited interrater reliability [13]. Intraoperative observation of a separate NI was taken from the surgeon’s documentation.

### Relationship to postoperative outcome

Relationship of traintime, tumor size and NN estimates of postoperative outcomes (postoperative and follow-up HB grades) with the actual observed outcomes was evaluated using Spearman partial rank correlation as applied previously [4]. A statistically significant partial correlation suggests an association which is not explained by the covariates, e.g. traintime and outcome independent of tumor size [4]. Evaluation of the correlation of only the raw traintime and tumor size with the outcome, i.e. without first passing through the networks was performed to yield a baseline performance to compare network outputs against.

### Neural networks and logistic regression models

Feed-forward networks with different input parameters, a single hidden layer and simultaneous postoperative and follow-up HB grades as outputs were constructed using the *feedforward* function of the Matlab Deep Learning Toolbox (Matlab R2021a, The Mathworks, Natick, MA, USA). Number of hidden layer neurons was chosen equal to the number of inputs. Continuous network outputs were rounded and interpreted as estimated HB grades. The networks therefore were trained to recognize the association between input parameters and the target “patterns” of HB grade pairs (postoperative and follow-up).

The procedure utilized a Levenberg-Marquardt training function and mean squared error for performance evaluation. Data was randomly separated into 75% (150 datasets) training and 25% (50 datasets) validation splits. Performance was evaluated in only the validation split by calculating chi^2^ statistic between estimated HB grades and postoperative and follow-up HB grades. For more intuitive interpretation, chi^2^ values were transformed into Cramér’s V effect sizes. For 5 × 5 tables (evaluated HB 1–5), values below 0.05 are considered negligible, 0.05–0.13 small, 0.13–0.22 medium and above 0.22 as large [14].

To illustrate the performance of a more transparent model, multivariable multinomial logistic regression models (LRM) were trained and evaluated with the feature combination showing the best NN performance, applying the same methodology.

### Statistical evaluation of performance

NN training depends on random choice of training and validation splits as well as random initialization of synapse weights between layers. To better estimate overall NN performance, we applied bootstrapping to sample the performance distribution observed with many networks. The approach repeated a single run of calculations 1000 times, yielding 1000 estimates, i.e., chi^2^ values of the comparison between network output and outcomes.

The mean and 95% confidence intervals of the resulting distribution was taken as overall performance. For calculation of significance, the distribution was compared to a surrogate distribution using a Komolgorov-Sminorv (KS) test. The surrogate distribution was constructed by shuffling input data of the validation in respect to the outcome values. Chi^2^ values were then calculated using surrogate network output. The procedure was also repeated 1000 times yielding the surrogate distribution.

### Comparison of different input sets

Primary endpoint of our study was to evaluate NN with inputs traintime, tumor size and preoperative facial nerve function. Additionally, we evaluated performance, when adding the information that a separate intermedius and/or A-train clusters were observed. Performance differences are discussed based on 95% confidence intervals (CI). Overlapping CI were interpreted as a lack of significant differences, which is considered conservative [15].

### Evaluation of tumor size

Networks trained on traintime, tumor size and preoperative facial nerve function were further analyzed to study the influence of tumor size. The complete dataset was subdivided into groups according to Koos grades. Chi^2^ values were then calculated for each group individually. Due to comparable preoperative HB grades in most patients and therefore also within tumor size subgroups, the observed group correlations then necessarily must depend on traintime. Mean correlations and 95%-CI are reported over all 1000 randomizations. For evaluation of differences between tumor size categories, a general linear regression model (GLM) was fitted to the network estimates, taking tumor size and sample size in the groups into account to control for the different patient numbers in tumor size groups, ranging from 18 with Koos 1 to 70 with Koos 3.

### Influence of a separate intermedius nerve

NN performance was investigated regarding the influence of a separate NI. Based on all 200 patients, chi^2^ of estimates and clinical HB grades were calculated for patients with and without separate NI in each of the 1000 randomizations and compared with the KS test. We decided not to perform this evaluation in only the validation split unlike the remaining analysis but in the complete sample. Due to the random selection of 50 cases in each randomization, this would have led to varying and frequently unbalanced percentages of cases with a separate NI. Since chi^2^ statistics and to some degree Cramér’s V are sensitive to the sample size, comparison to performance of other neural networks evaluated in only the smaller validation split is limited.

## Results

### Patients

Mean age of the 200 included patients was 51 years (21–80 years). 109 patients were women. Tumor size was Koos 1 in 18 patients, Koos 2 in 57, Koos 3 in 70 and Koos 4 in 55 patients [16]. Preoperative facial nerve function was HB 1 on median (range 1–3, 3 patients with HB 3) [12]. A separate NI was observed intraoperatively in 99 patients.

### Conventional analysis

**Fig. 1:**
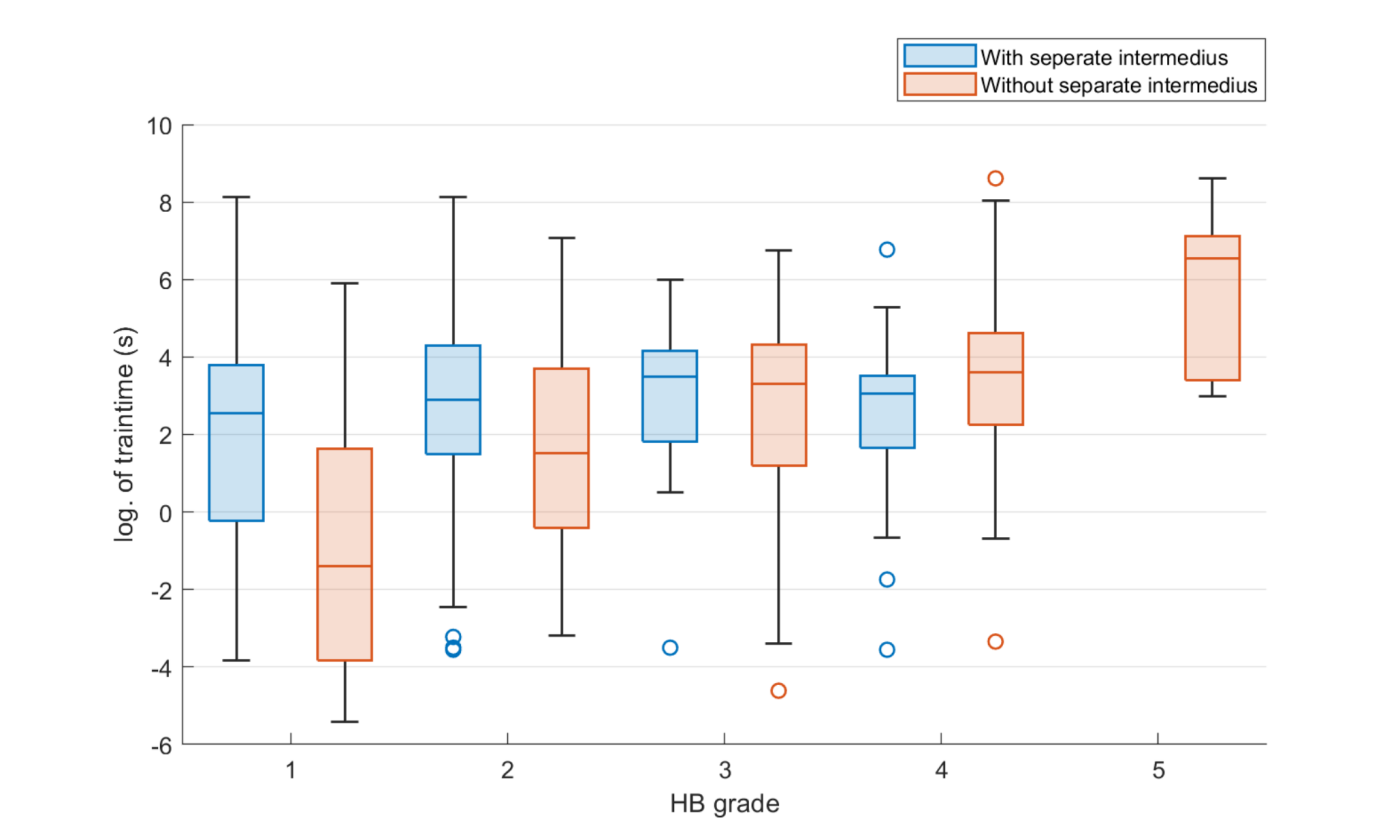
Mean traintime over all channels in patients with and without a separate intermedius nerve

Table 1 provides an overview of results. Traintime was significantly correlated to postoperative and follow-up HB. In patients without separate NI, correlations were higher than in patients with separate NI. A-train clusters were more frequently observed in patients with a separate intermedius (Fig. 1). However, removal of A-train clusters resulted in only negligible improvement in the complete group.

**Table 1:** Correlations with postoperative and follow-up HB of conventional analysis. Significant correlations are printed in bold

Group	Parameters	HB	Spearman correlation	
			**rho**	**p**
All patients	Mean traintime	Postop.	**0.397**	**<0.0001**
		Follow-up	**0.323**	**<0.0001**
	Mean traintime without clusters	Postop.	**0.442**	**<0.0001**
		Follow-up	**0.350**	**<0.0001**
	Tumor size (Koos)	Postop.	**0.456**	**<0.0001**
		Follow-up	**0.437**	**<0.0001**
	Mean traintime, tumor size controlled (partial correlation)	Postop.	**0.208**	**<0.005**
		Follow-up	0.123	0.084
Without sep. intermedius nerve	Mean traintime	Postop.	**0.564**	**<0.0001**
		Follow-up	**0.511**	**<0.0001**
	Tumor size (Koos)	Postop.	**0.564**	**<0.0001**
		Follow-up	**0.509**	**<0.0001**
With sep. intermedius nerve	Mean traintime	Postop.	**0.198**	**0.0499**
		Follow-up	0.08	>0.1
	Tumor size (Koos)	Postop.	**0.317**	**<0.0001**
		Follow-up	**0.341**	**<0.0001**

**Fig. 2:**
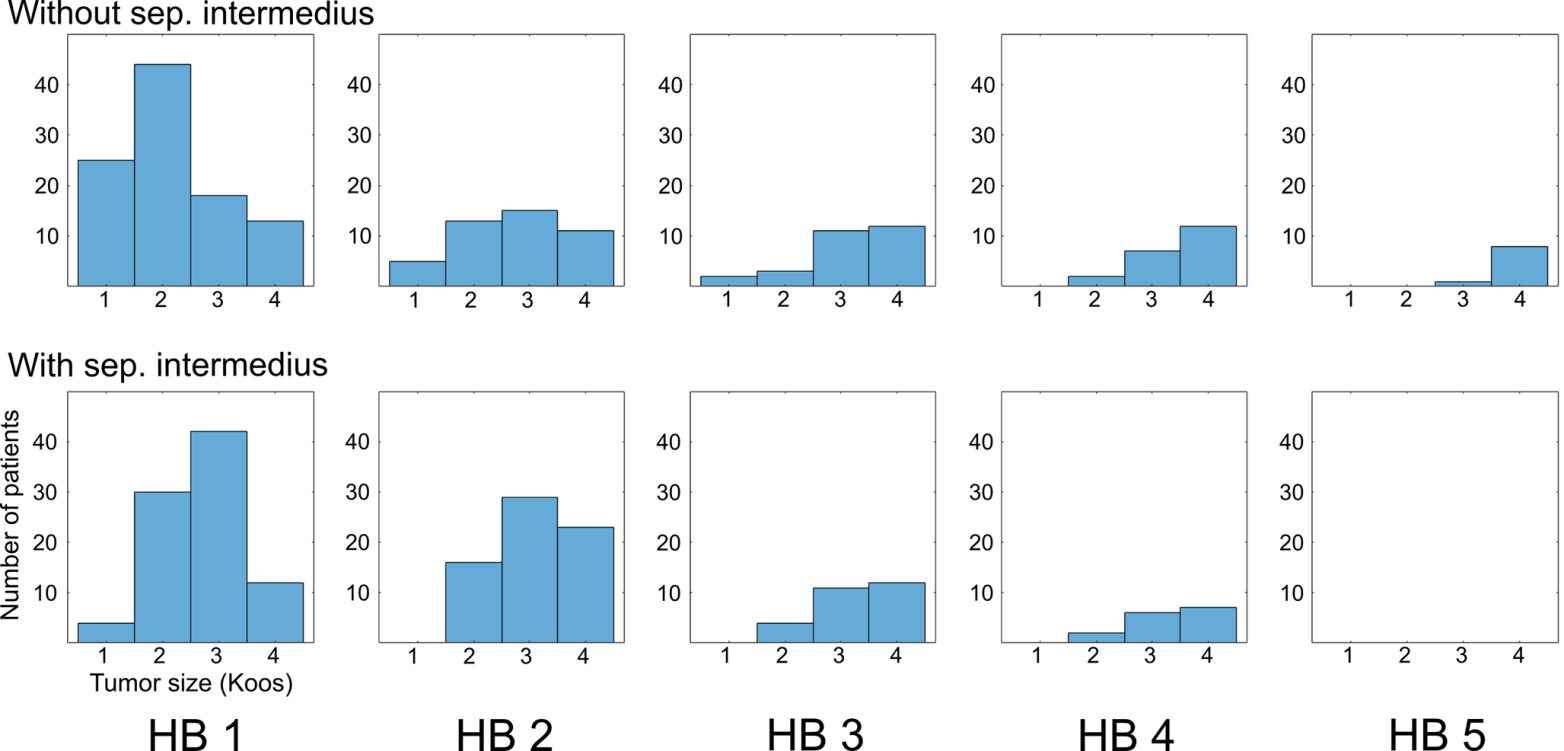
Koos tumor size in relation to facial nerve outcome. Postoperative and follow-up are pooled

Tumor sizes also correlated with outcomes and patients with a separate NI had larger tumors than patients without (p = 0.0012, chi^2^ = 15.96, chi-square test). In patients with a separate NI, correlations of tumor size with facial nerve function were lower compared to cases without (Fig. 2).

Controlling for tumor size, partial correlations yielded significant remaining correlations for immediate postoperative traintime and facial nerve function, but were not significant at follow-up.

### Neural networks and LRM

**Fig. 3:**
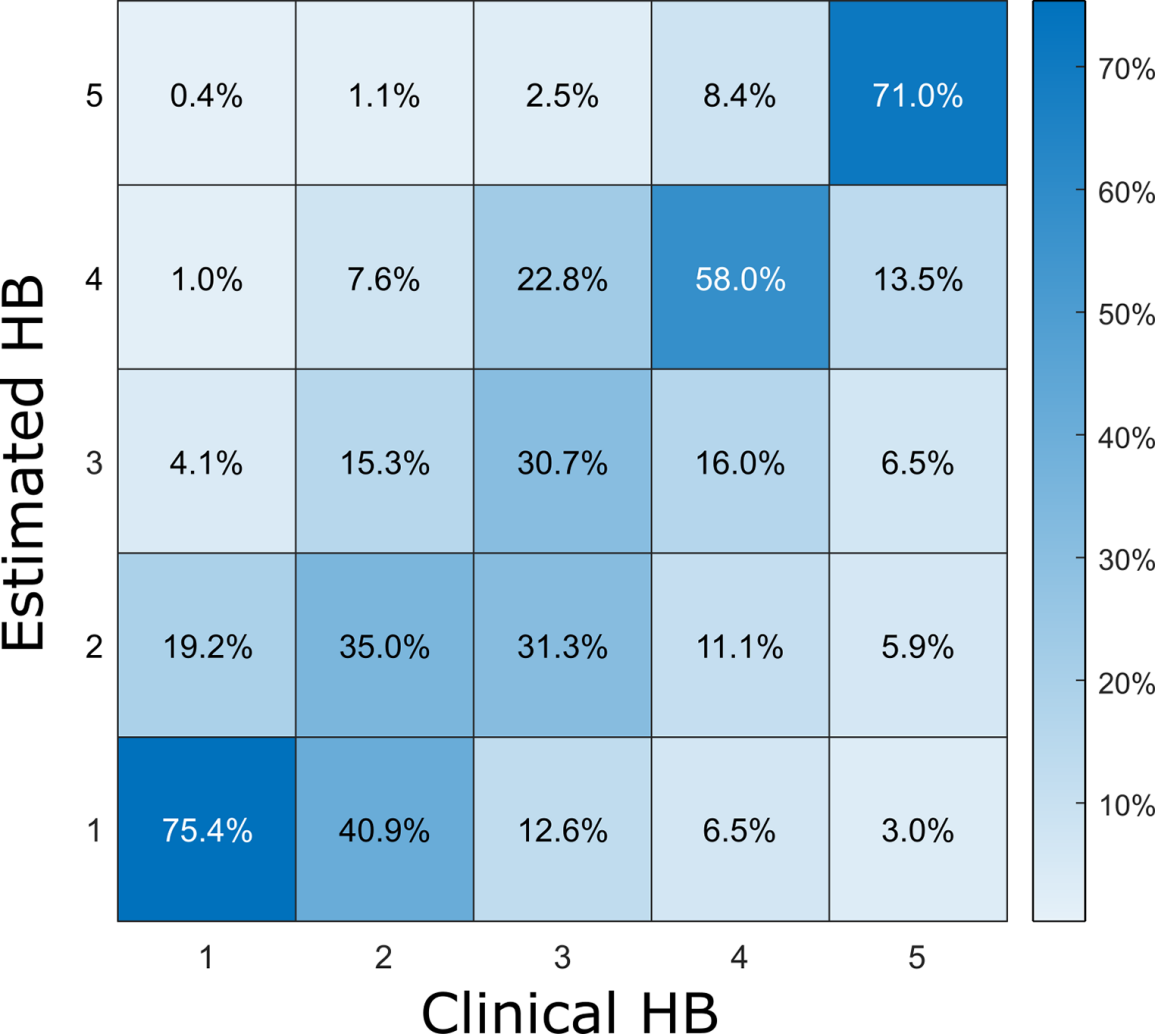
Concordance of clinical HB grades and neural network estimates of the network with inputs yielding the best results (Traintime without clusters, Koos, preoperative HB-grade). Coloring and percentages in each (independent) column give the portion of all randomized results with a specific HB grade. For example, 75.4% of neural estimates in cases with postoperative or follow-up HB 1 also suggest HB 1, while 19.2% suggest HB 2 and thus overestimate facial nerve palsy.

Using traintime, tumor size and preoperative HB grades as input, mean chi^2^ comparing NN estimates and outcomes was chi^2^ = 51.3 (p < 0.0001) corresponding to a Cramér’s V of 0.36 evaluated only in the validation split. Tables 2 and 3 as well as Fig. 3 show results in detail.

Using the observation of a separate NI or A-train clusters as additional inputs yielded comparable results. Performance using only tumor size or only mean traintime over all channels yielded considerably lower results.

**Table 2:** Performance of neural network estimates. Results in the validation split (50 patients) are reported

Inputs	Chi^2^		Cramér’s V
	**mean**	**CI**	
Koos only	30.6	30.1-31.1	0.28
Mean traintime only	31.9	30.6-33.2	0.28
Mean traintime only (without clusters)	47.7	45.9-49.5	0.35
Traintime, Koos, preOP HB	51.3	49.7-53.0	0.36
+ sep. intermedius	49.1	47.6-50.7	0.35
+ A-train cluster	49.7	48.1-51.3	0.35
+ sep. intermedius and A-train cluster	44.8	43.4-46.2	0.33
Traintime (without clusters), Koos, preOP HB	54.8	53.0-56.7	0.37
+ sep. intermedius	51.6	50.0-53.2	0.36
+ A-train cluster	52.1	50.4-53.8	0.36
+ sep. intermedius and A-train cluster	49.0	47.4-50.7	0.35

All input combinations were reevaluated using traintime with manually removed A-train clusters (“corrected traintime”). This resulted in a considerable improvement even when only mean traintime over all channels was used as input. Networks with tumor size, preoperative HB and corrected traintime values also resulted in a mean higher chi^2^ value.

**Table 3:** Performance of neural network estimates in tumor size subgroups. Training was performed with traintime, tumor size and preoperative facial nerve function. Results were then subdivided according to Koos grade for calculation of chi2 and Cramér’s V

Tumor size	Chi^2^		Cramér’s V
	**mean**	**CI**	
Koos 1	4.8	4.7-5.0	0.08
Koos 2	35.4	34.5-36.0	0.21
Koos 3	118.6	115.2-122.1	0.39
Koos 4	61.3	60.1-62.5	0.28

Using the combination of features with best neural network performance as inputs to LRMs (traintime without clusters, Koos, preoperative HB, chi^2^ = 54.8, Table 2), yielded a lower mean chi^2^ of 41.4 (confidence interval 40.9–41.9), corresponding to a Cramér’s V of 0.37.

### Tumor size

Analysis of concordance with postoperative facial nerve function in Koos subgroups are presented in Table 3. Differences of chi^2^ values between groups reached statistical significance, also after correcting for the expected tumor and sample size interaction (GLM analysis, F = 2380, p < 0.0001 for the regression model, t = -40.8, p < 0.0001 for factor tumor size).

### Influence of a separate intermedius nerve

Comparison of performance in all patients yielded significantly better values in patients without a separate NI using the best set of inputs (preoperative HB, tumor size and corrected traintime): chi^2^ = 164.2 vs. 65.9 (p < 0.0001), corresponding to a Cramér`s V of 0.46 (n = 99 patients) and 0.29 (n = 101 patients). Networks utilizing corrected traintime showed improved performance only in patients without a separate NI (best chi^2^ with A-train clusters: 32.7 vs. 35.6 without and 18.3 vs. 17.0 with a separate NI, Table 4).

**Table 4:** Comparison of neural network performance in patients with and without a separate intermedius nerve. Results in the validation split are reported, grouped according to intraoperative observation of a separate intermedius nerve. Due to the lower sample number in each group (on average approx. 50% due to the portion of patients with separate intermedius nerve), chi^2^ and Cramér´s V are generally lower compared to Table 1

Inputs	With sep. intermedius nerve			Without sep. intermedius nerve		
	**Chi** ^**2**^		**Cramér’s V**	**Chi** ^**2**^		**Cramér’s V**
	**mean**	**CI**		**mean**	**CI**	
Koos only	14.0	13.7-14.4	0.13	21.5	21.0-21.9	0.16
Mean traintime only	10.1	9.7-10.4	0.11	24.3	23.3-25.2	0.17
Mean traintime only (without clusters)	10.3	10.0-10.6	0.11	33.3	32.2-34.5	0.20
Traintime, Koos, preOP HB	18.3	17.8-18.8	0.15	32.7	31.7-33.6	0.20
+ sep. intermedius	17.6	17.1-18.1	0.15	31.9	31.0-32.8	0.20
+ A-train cluster	17.5	17.0-17.9	0.15	33.0	32.0-34.0	0.20
+ sep. intermedius and A-train cluster	15.9	15.4-16.4	0.14	30.6	29.8-31.5	0.19
Traintime (without clusters), Koos, preOP HB	17.0	16.5-17.5	0.15	35.6	34.5-36.7	0.21
+ sep. intermedius	16.7	16.2-17.2	0.15	35.1	34.1-36.0	0.21
+ A-train cluster	16.7	16.2-17.2	0.15	35.2	34.1-36.2	0.21
+ sep. intermedius and A-train cluster	15.5	15.0-16.0	0.14	33.3	32.3-34.2	0.20

## Discussion

We utilized machine learning approaches in a group of 200 patients undergoing VS surgery. Our results show that these methods can combine preoperative facial nerve function, tumor size and intraoperative traintime to estimate postoperative facial nerve outcomes. Performance exceeds results from evaluation of the features alone and when tumor size is controlled. Performance did not improve when observation of a separate NI and/or detection of A-train clusters were added to the analysis. Prediction improved when A-train-clusters were removed from the detected traintime, mainly due to improvements in patients without a separate NI. Improved prediction may support intraoperative decision making as well as recognition, which surgical maneuvers carry an increased risk for postoperative palsy.

Our previous studies demonstrated that a separate NI can give rise to an exceeding amount of A-trains not related to postoperative palsy [6, 11], which limits outcome estimation based on free-running EMG alone. Since observation of a separate intermedius is related to tumor size [11], which itself yields predictive information [4, 17, 18], we hypothesized that considering this interaction could improve outcome estimation.

Indeed, integrating preoperative facial nerve function, traintime and tumor size outperformed outcome estimation using only tumor size or traintime. Although performance was generally lower in patients with a separate NI, combined analysis also resulted in improvements in this subgroup.

Preoperative facial nerve function and tumor size have been shown to impact intraoperative monitoring. Facial MEP for example correlate with tumor size already at the start of surgery [19], while traintime interpretation should consider preoperative deficits [3]. Our results show that NN approaches integrate these different modalities, effectively implementing such clinical recommendations in a formalized and objective manner.

Utilizing corrected traintime resulted in a considerable improvement even if only mean traintime was considered. Correction increased chi^2^ from 31.9 to 47.7 (Cramér`s V from 0.39 to 0.49). The combination with preoperative HB and tumor size then showed the best of all tested combinations. Correction was based on our previous findings, that patients with separate NI show A-train clusters significantly more often than patients without [11], similar to patients with previous surgery or irradiation [5]. We argued that these clusters are an expression of a hyperexcitable or more vulnerable NI.

The result that removing A-train clusters is beneficial for HB estimation supports the idea that such excessive, clinically not informative traintime may be caused by a separate NI [6, 11]. It is however surprising that considering the observation of a separate intermedius or the presence of clusters to NN was not helpful and even partially decreased performance. Furthermore, the effect was largely present in the subgroup without separate NI, while patients with NI did not benefit (Table 4).

Consequently, the results indicate that A-train clusters generally over-represent actual damage to the facial nerve – not only when a split nerve course is encountered. Cluster traintime should therefore be weighted weaker than traintime from singular A-trains or removed entirely. In the current study, correction however was not sufficient to ameliorate the impact of a separate NI. There are several potential reasons. First, due to practical factors, A-train clusters were identified visually. This strategy may have resulted in marking only the clearest of clusters, while the phenomenon might in fact be subtler and manifest as a “spectrum of over-representation”. Furthermore, topography, time and distance between occurrences and relationship to singular A-trains were not evaluated.

Even if such information would not alleviate the intermedius “issue”, NN offer further potential improvement. NN allow integration of more information sources, above and beyond the evaluated features. E.g., FMEP [19–21] or direct electrical stimulation [22] could be utilized for a multimodal monitoring approach. In addition, determination of the facial nerve course [23] could add valuable anatomical information.

Overall, estimated HB grades corresponded well to clinical evaluation. In moderate ranges, we observed deviations by one, sometimes two degrees (Fig. 3). Such variability may partially be caused by the subjective nature of HB grading itself, respectively its practical application [24–26]. Scheller et al. [13] investigated the interobserver variability of HB grading as part of a randomized multi-center phase III trial. In this study, too, HB grades varied between observers in an extent comparable to our results. HB grades were also most consistent when facial nerve function was normal or mildly impaired. NN estimates are therefore well within the range of this variability. Further improvement may require the use of a more objective grading system with better interrater reliability [26–29].

Finally, a significant disadvantage of neural network is their “black box” nature, i.e., how they achieve their performance is notoriously difficult to interpret. In comparison, LMR are more accessible, as the resulting regression coefficient allow direct interpretation of the relative feature importance. The performance of LMRs in our study was lower than with NN, however still within a clinically useful range. It is conceivable that more training data may result in further improvement. Future studies should therefore conduct more detailed comparisons, including further computational approaches to combine multimodal information.

## Conclusion

In conclusion, NN using traintime, preoperative facial nerve function and tumor size can estimate postoperative HB grades with good accuracy. However, they do not fully compensate false positive A-train activity associated with a separate NI. Removal of A-train cluster traintime nevertheless seems to be advisable even in cases without a separate course of the intermediate nerve. NN can integrate information from different pre- and intraoperative diagnostic methods and may enable comprehensive multimodal monitoring.
