# Correction to: Neural networks for estimation of facial palsy after vestibular schwannoma surgery

**DOI:** 10.1007/s10877-022-00950-x

**Published:** 2022-12-08

**Authors:** Stefan Rampp, Magdalena Holze, Christian Scheller, Christian Strauss, Julian Prell

**Affiliations:** 1 Department of Neurosurgery, University Hospital Halle (Saale), Ernst-Grube Str. 40, 06120 Halle, Germany; 2 Department of Neurosurgery, University Hospital Erlangen, Schwabachanlage 6, 91054 Erlangen, Germany

**Correction: Journal of Clinical Monitoring and Computing (2022)**

10.1007/s10877-022-00928-9.

In this article the title was incorrectly given as “Original Research” instead research Neural networks for estimation of facial palsy after vestibular schwannoma surgery’ but should have been ‘Neural networks for estimation of facial palsy after vestibular schwannoma surgery’.

The original article has been corrected.

